# Protective antibody and cytokine responses in mice following immunization with recombinant beta-tubulin and subsequent *Trypanosoma evansi* challenge

**DOI:** 10.1186/s13071-015-1189-3

**Published:** 2015-11-09

**Authors:** Anup Kumar Tewari, Samarchith P. Kurup, Surajit Baidya, John R. Barta, Bhaskar Sharma

**Affiliations:** Department of Pathobiology, Ontario Veterinary College, University of Guelph, Guelph, ON N1G 2W1 Canada; Division of Parasitology, Indian Veterinary Research Institute, Izatnagar, Uttar Pradesh 243 122 India; Department of Microbiology, University of Iowa Carver College of Medicine, Iowa City, IA 52242 USA; Division of Animal Biochemistry, Indian Veterinary Research Institute, Izatnagar, Uttar Pradesh 243 122 India; Department of Parasitology, West Bengal University of Animal and Fishery Sciences, 37 & 68, Kshudiram Bose Sarani, Belgachia, Kolkata, West Bengal 700037 India

**Keywords:** Recombinant beta tubulin, Cytokine, Immune response, Surra, *Trypanosoma evansi*

## Abstract

**Background:**

Trypanosomosis or Surra, caused by the flagellated hemoprotozoan parasite *Trypanosoma evansi*, is a disease of economic importance through its wide prevalence in domestic livestock in tropical countries. In the absence of a protective vaccine, management of the disease relies on a few available chemotherapeutic agents. Although humoral immunity is the mainstay of resistance to *T. evansi*, the ability of the parasite to vary its immunodominant surface proteins to subvert the immune system has forced vaccine efforts to target a variety of invariant epitopes. Beta tubulin, an integral component of the trypanosome cytoskeleton, was therefore targeted using the recombinant form of the protein for immunization.

**Methods:**

The 1329 bp coding sequence of beta tubulin gene was PCR amplified and cloned in pQE-TriSystem expression vector. Recombinant beta tubulin was heterologously expressed in *Escherichia coli* as a 46 KDa fusion protein and used for immunization of mice. The Ig response was studied by ELISA, whereas the cytokine response was measured using a cytometric bead-based assay quantified by flow cytometry.

**Result:**

Immunization with recombinant beta (β)-tubulin protein induced a beta-tubulin specific humoral immune response of predominantly IgG2a isotype. Lethal challenge with *T. evansi* blood-form trypomastigotes post-immunization elicited a robust anamnestic response. An abundance of IFN-γ further confirmed the Th-1 bias of the protective response. We also observed extended survival and better control of the challenge infection in the immunized mice.

**Conclusions:**

A robust anamnestic response following challenge including a Th-1 serum cytokine profile coupled with increased survival is indicative of protective immunity in the immunized mice. These observations suggest that β-tubulin of *T. evansi* is a viable antigenic target for development of a vaccine against this important livestock pathogen.

## Background

*Trypanosoma evansi*, a unicellular hemoflagellate, is the causative agent of ‘surra’, a debilitating disease of a wide range of livestock species in the tropics. This parasite is transmitted mechanically among its wide range of susceptible hosts by several species of hematophagous flies in Asia, Africa, Latin America and parts of Europe [1–4]. The progression and outcome of infections varies widely depending on the host species; fever, progressive anemia or corneal opacity is typical in companion animals whereas farm animals suffer from loss of condition, productivity and draught efficiency, neurological abnormalities, abortion or immunosuppression [5–8].

Management of surra is based chiefly on chemotherapy. However, limited therapeutic options and the development of drug-resistance have posed a major threat to our abilities to contain the disease. The need for a safe and cost-effective vaccine against surra [9] makes identification of protective epitopes a priority. Though *T. evansi* has an abundance of highly immunogenic glycoproteins presented on its surface (variable surface glycoprotein, VSG), the active and systematic variation of these surface antigens during the course of an infection has limited the prospects of targeting them for vaccine development [10]. Consequently, immunization strategies in surra have focused on various alternate, invariant, and often immunologically subdominant epitopes [11–14].

Intracellular antigens can act as protective epitopes in parasitic infections including malaria [15], schistosomosis [16] and trypanosomosis [9, 10, 12] although the underlying mechanisms have not been described. One promising intracellular antigen of trypanosomes is beta (β) tubulin which belongs to the large tubulin protein family. Unlike in higher vertebrates and other invertebrates that possess orthologues of β tubulin with diverse and dissimilar structural and functional attributes, trypanosomes have a single isoform in the Trypanosomatidae [10]*.* This protein is an important structural component of the flagellum and is critical for structural stability and replication making it a promising therapeutic and vaccine target in protozoa [9]. Immunization with native, recombinant or cDNA encoded trypanosomatid tubulin has shown varying levels of protection against different African trypanosomes in experimental surra [9, 10, 17].

Humoral immunity has long known to be important in protection against African trypanosomes. Induction of a rapid B-cell activation [18, 19] accompanied by a T helper type (Th) 1 response with production of pro-inflammatory cytokines is characteristic of successful immunological control of acute African trypanosomosis in the host [20–23] . A fine balance between the pro-inflammatory and anti-inflammatory cytokines that effectively controls the parasitemia [24] and associated anemia [25] in newly acquired infections are thought to be responsible for trypanotolerance. In the present study, we characterized the humoral antibody responses and associated Th1-polarized serum cytokine profiles in mice following immunization with recombinant *T. evansi* β-tubulin that provided enhanced survival of mice in the face of a lethal challenge infection.

## Methods

### Mice

Female Swiss albino mice (6–8 weeks) were procured from the Laboratory Animal Research (LAR) Division, Indian Veterinary Research Institute (IVRI), Izatnagar. The mice were maintained under standard feeding and rearing conditions at the lab animal housing facility of the Division of Parasitology, IVRI, Izatnagar.

### Recombinant expression-vector

The entire open reading frame (ORF) of *T. evansi* β-tubulin (EU483116) was amplified by PCR from *T. evansi* cDNA template with primers, F_1_X: 5′-AGAATTCCATGGGTGAGATTGTGTGCGT-3′ containing *Nco*1 restriction site and R_1_X: 5′-TGAATTCTCGAGGTATTGCTCCTCCTCGTC-3′ containing the *Xho*1 restriction site. The purified PCR product was digested with *Nco*1 and *Xho*1 and cloned into pQE TriSystem plasmid vector (Qiagen, CA, USA) to give pQE-*Te-*β-tubulin recombinant plasmid.

### Expression of r*Te-*β-tubulin

Recombinant pQE-*Te-*β-tubulin construct was used for expression of *Te-*β-tubulin in *E. coli* as per the manufacturer’s protocol (Qiagen, CA, USA). In short, 100 μl of overnight culture of pQE-*Te-*β-tubulin transformed M15 strain *E. coli* was transferred into 50 ml of LB broth (Thermo Scientific, MA, USA) containing 100 μg/ml ampicillin and was allowed to grow in a shaking incubator at 37 °C until mid-log phase (~2 h) after which isopropyl-beta-D-thiogalactopyranoside (IPTG) (Thermo Scientific, MA, USA) was added at 1 mM final concentration to induce expression. The culture was maintained for up to 6 h post induction. r*Te-*β-tubulin produced in the *E. coli* cell lysate screened using SDS-PAGE. The identity of the expressed protein was confirmed by western blot probed with anti-histidine (His) tag antibody (Invitrogen, CA, USA). The His-tagged r*Te-*β-tubulin present in the insoluble pellet was solubilized under denaturing conditions with 8 m urea, and then purified using Ni-NTA agarose column (Qiagen, CA, USA), renatured by dialysis (MWCO 10 K, Thermo Scientific, MA, USA) against PBS, pH7.4, at 4 °C for 36 h and its purity determined by SDS-PAGE and stored at −20 °C until use. The concentration of r*Te-*β-tubulin was determined with modified Lowry protein assay kit (Thermo Scientific, MA, USA) as per the manufacturer’s protocol.

### Immunization and challenge

Female Swiss albino mice (6–8 weeks) were immunized subcutaneously with 50 μg of r*Te-*β-tubulin in Freund’s complete adjuvant (FCA) or with FCA alone as control. Similar booster dosages were administered 21 days later, but with incomplete Freund’s adjuvant (IFA). At 35 days post primary immunization, all mice were challenged intraperitoneally with 10^3^ *T. evansi* blood form trypomastigotes.

### Measurement of specific antibody response

The serum antibody response was studied by enzyme linked immunosorbent assay (ELISA). A standard 96 well ELISA plate (Nunc, NY, USA) was coated for 1 h at 37 °C with the r*Te-*β-tubulin (5 μg/ml dissolved in 50 mM sodium carbonate coating buffer, pH 9.6) followed by overnight blocking at 4 °C with 5 % bovine serum albumin in PBS. After washing, 100 μl of heat inactivated serum (56 °C for 30 min) from immunized or naive mice was added at 1:50 dilution. After incubating for 2 h at 37 °C, sera samples were removed, the wells washed and 100 μl of HRPase conjugated goat anti-mouse IgG (Bangalore Genie, Karnataka, India) or IgG2a (Santa Cruz, CA, USA) added at 1:5000 dilution to the wells and incubated for 2 h at 37 °C. Washed plates were developed for 15 mins at RT, using 100 μl/ well of OPD substrate (15 μg/ml in citrate phosphate buffer, pH 5.0). The reaction was stopped by the addition of 50 μl 2 M sulfuric acid and the plates were read at OD_492nm_ in a micro plate reader (Tecan, Mannedorf, Switzerland).

### Measurement of serum cytokines

The concentration of Interleukin (IL)-4, IL-5, IL −10, Interferon (IFN)- γ and Tumor Necrosis Factor (TNF)-α were determined in the serum of mice every 24 h until 6 days post *T. evansi* challenge using the BD mouse Th1/Th2 cytokine CBA Kit (BD Biosciences, CA, USA) following the manufacturer’s protocol. Briefly, 50 μl of diluted serum sample separated from blood collected every 24 h post challenge, until 6 days was mixed with 50 μl of the mixed capture beads and 50 μl of the mouse Th1/Th2 PE detection reagent. The tubes were incubated at room temperature for 2 h in the dark, followed by a wash. The samples were subsequently resuspended in 300 μl of wash buffer before acquisition on the FACS Calibur Flow Cytometer (BD Biosciences, CA, USA) and analyzed using the FCAP Array Software (BD Biosciences). Standard curves were generated for each cytokine using the mixed bead standard provided with the kit and were used to determine the concentration of each cytokines in the serum samples. Serum IL-10 level was determined using an ELISA based kit (Thermo Scientific, MA, USA) following the manufacturer’s protocol.

### Determination of parasitemia

The parasitemia in mice following *T. evansi* challenge was monitored microscopically every 24 h from day 1 until 11 post infection using a standardized dilution of blood obtained from tail bleeding. Counts are expressed as trypomastigotes per 40× microscopic field and data are presented as means for each time point.

### Statistical analysis

Data are expressed as mean ± standard error of mean (s.e.m) and are derived from at least triplicate observations per sample per time-point. Results were analyzed by Student *t*-test and differences were considered significant if the *p*- value was ≤ 0.05.

### Ethics statement

All animal experimentation was conducted in compliance with the ethical considerations and guidelines issued by the Committee for the Purpose of Control and Supervision of Experiments on Animals (CPCSEA) of the Government of India and with the approval of the Institutional Animal Ethics Committee (IAEC, IVRI) regarding laboratory animals.

## Results

### Heterologous expression and purification of *Te-*β-tubulin

**Fig. 1:**
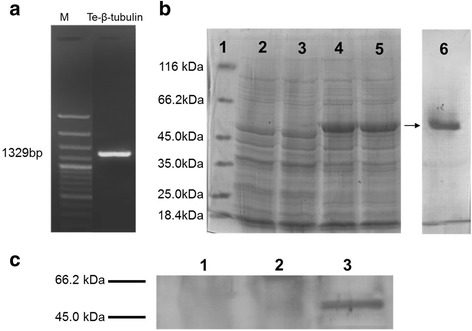
Heterologous expression of recombinant β-tubulin from *T. evansi*. **a** PCR amplification of *T. evansi* β-tubulin gene. **b** Induced heterologous expression and purification of recombinant *T. evansi* β-tubulin protein (50 kDa) shown by SDS-PAGE. Protein molecular weight marker (Lane 1), pre-induction *Te-*β-tubulin transgenic *E. coli* lysate (lane 2), induced *Te-*β-tubulin transgenic *E.coli* lysate collected 2, 4,or 6 h post-induction (lanes 3-5, respectively) and affinity-purified, renatured r*Te-*β-tubulin (lane 6). **c** Identity of the expressed r*Te-*β-tubulin confirmed by Western blot using anti-His-tag antibody probing induced (4 h) *Te*-β-tubulin transgenic *E. coli* culture supernatant (lane 1), pre-induction (lane 2) or induced (4 h) (lane 3) *Te-*β-tubulin transgenic *E. coli* lysate

To generate r*Te-*β-tubulin, the amplified *Te-*β-tubulin gene (Fig. 1a) from a cDNA template of *T. evansi* trypomastigote stage was first cloned into the expression inducible pQE-TriSystem vector. *Escherichia coli* transformed with this construct expressed r*Te-*β-tubulin that was purified by affinity column chromatography (Fig. 1b). The identity of expressed r*Te-*β-tubulin protein was confirmed by Western blot (Fig. 1c).

### Serum cytokine and antibody responses following immunization

**Fig. 2:**
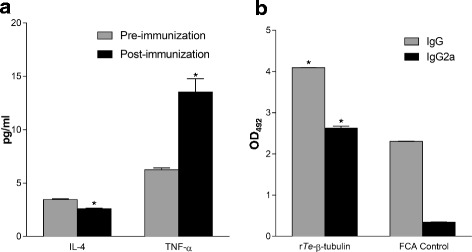
*Trypanosoma evansi* β-tubulin-specific immune response induced post immunization with r*Te-*β-tubulin. **a** Serum levels of IL-4 and TNF-α in mice pre- and 24 h post-inoculation with FCA adjuvenated r*Te-*β-tubulin; asterisks indicate significant differences (*p* ≤ 0.05) between the pre- and post-immunization mean levels for each cytokine. **b** *Te-*β-tubulin-specific IgG and IgG2a antibody in mouse sera at 14 days post immunization with r*Te-*β-tubulin or FCA control. Data presented as mean ± s.e.m. from at least 6 mice/treatment; asterisks indicate significant differences (*p* ≤ 0.05) between the mean OD_492_ values for serum IgG or IgG2a of FCA adjuvenated r*Te-*β-tubulin vaccinated mice compared with FCA-only inoculated control mice

To determine cytokine responses following vaccination and subsequent specific humoral immune response mounted against r*Te-*β-tubulin, sera from mice inoculated with r*Te-*β-tubulin (50 μg) in FCA or FCA alone (control) were compared. At 24 h post inoculation, mice inoculated with r*Te-*β-tubulin in FCA showed significantly lower IL-4 and significantly higher TNF-α levels in the serum compared to pre-immunization levels (Fig. 2a). IL-4 or TNF-α levels in mice inoculated with FCA alone did not differ significantly from the mice inoculated with r*Te-*β-tubulin in FCA (data not shown). At 14 days post inoculation, mice immunized with r*Te-*β-tubulin had developed significantly higher titers of *Te-*β-tubulin-specific IgG antibodies (1.8 fold higher OD_492_ readings) compared to FCA controls; *Te-*β-tubulin-specific IgG2a titers in vaccinated mice were significantly higher (7.7 fold higher OD_492_ readings) than FCA control mice (Fig. 2b).

### Serum cytokine and antibody responses following *T. evansi* challenge

**Fig. 3:**
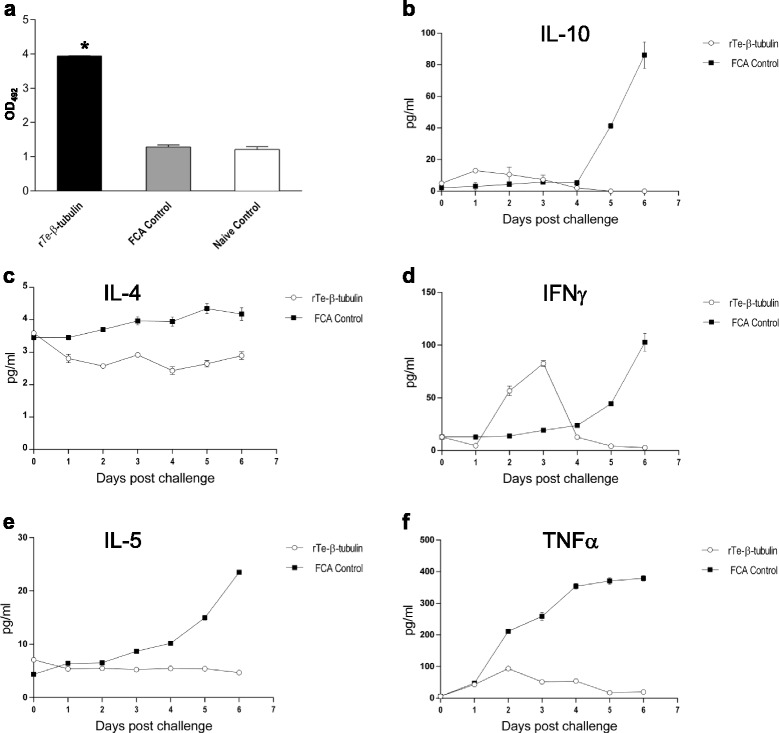
*Trypanosoma evansi* β-tubulin specific recall response with Th1 polarization in lethal challenge of r*Te-*β-tubulin immunized mice. **a** Higher r*Te-*β-tubulin specific (IgG) antibody titers in sera of mice immunized with r*Te-*β-tubulin and challenged with *T. evansi* blood stream forms; asterisk indicates significant difference (*p* ≤ 0.05) between the mean OD_492_ value for serum IgG in the serum of r*Te-*β-tubulin immunized mice compared with adjuvant and infection controls. **b**-**f** Kinetics of the serum levels of IL-4 (**b**), IL-5 (**c**), IFN-γ **d** and TNF-α (**e**) in r*Te-*β-tubulin or the adjuvant control immunized mice post challenge with *T. evansi* blood stream forms. Data presented as mean ± s.e.m. from at least 6 mice/treatment

Mice challenged with a lethal dose of live blood stage *T. evansi* 35 days post-immunization showed significantly higher *Te-*β-tubulin specific (IgG) serum antibody titers compared to adjuvant-control or unimmunized mice at 4 days post-challenge (Fig. 3a). Mice inoculated with r*Te-*β-tubulin in FCA and challenged with *T. evansi* 35 days post-immunization had dramatically different kinetics of serum cytokine levels compared with mice inoculated with FCA only (Figs. 3b-f). Mice immunized with r*Te-*β-tubulin demonstrated unchanging or slowly dropping serum IL-4 and IL-5 concentrations following challenge. Modest early (1–2 DPC) increases in the concentration of TNF-α and IL-10 were followed by slowly decreasing serum concentrations in mice inoculated with r*Te-*β-tubulin in FCA. In these same mice, interferon-γ increased in concentration following challenge (peaking at 3 DPC) and then returned to lower levels for the remainder of the challenge period. In contrast, all measured cytokines increased from their levels at day of challenge until the end of the challenge period in mice inoculated with FCA only.

### r*Te-*β-tubulin immunization enhanced survival following *T. evansi* challenge

**Fig. 4:**
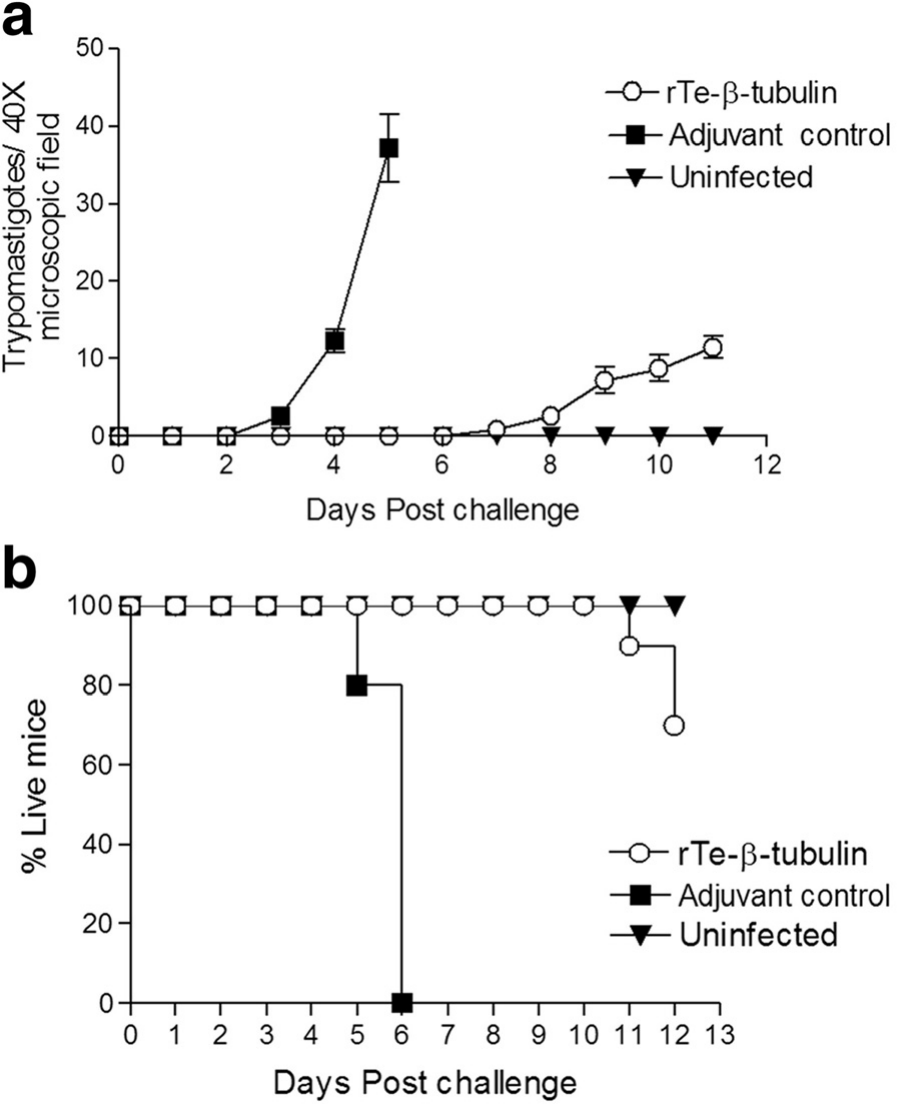
Mice immunized with r*Te-*β-tubulin are better protected from a lethal challenge with *T. evansi*. The r*Te-*β-tubulin immunized mice demonstrated lower parasitemia (**a**) and prolonged survival (**b**) following a virulent *T. evansi* challenge compared with similarly challenged adjuvant only controls. Daily counts of bloodstream trypomastigotes represent means ± s.e.m. from at least 10 mice per treatment

Mice immunized with r*Te-*β-tubulin exhibited a delayed appearance, as well as lower numbers, of *T. evansi* trypomastigotes in blood following challenge (Fig. 4a). All mice in the FCA only control group died by day 6 post challenge whereas all mice immunized with r*Te-*β-tubulin in FCA survived until at least day 10 post challenge. However, all the mice in the latter group eventually succumbed to infection by 28 days post challenge (Fig. 4b).

## Discussion

Surra is an insidious disease complex caused by the unicellular, flagellated protozoan *T. evansi* that leads to the development of severe and often fatal anemia. The infection is prevalent in a wide range of domestic and wild animal species across the tropical and semitropical regions of Eurasia and Africa [8]. Although management of the disease by chemotherapy is feasible, its successful application is limited by the low sensitivity of conventional microscopy and serological tools that prevent the timely identification of chronic infections and carrier animals. The spread of the infection in susceptible animals often remains unchecked impacting the health and productivity of livestock in the tropics. The ability of *T. evansi* to vary its immuno-dominant major surface antigen to escape host immune responses limits the prospects of developing a conventional vaccine against *T. evansi* targeting epitopes typically exposed on the plasma membrane of the parasite [10]. Consequently, evaluation of internal antigens of *T. evansi* (e.g. molecules not typically exposed on the cell surface) for their ability to induce protective immune responses has become necessary [10, 17].

There have been multiple attempts at developing native or recombinant protein based vaccines against various protozoal infections but often with limited success compared vaccines based on whole organisms that better reflect the complexity of the target pathogen [26, 27]. Protective immunization against African trypanosomosis has been particularly challenging owing to the sequential expression of variable surface antigen molecules by the parasite that efficiently subverts the humoral immune responses [14, 28]. However, induction of protective immunity to various levels has been achieved by targeting invariable antigens on African trypanosomes; mice vaccinated with native tubulin purified from *T. b. brucei* were protected from *T. b. brucei, T. congolense* or *T. b. rhodesiense* infections [9]. Likewise, immunization with the microtubule associated protein (MAP) p52, GAPDH and MAP p15 protected mice from *T. brucei* challenge [29]. Recombinant protein [10, 12] or DNA [17] based vaccines targeting the tubulin of various African trypanosomes have also provided partial protection.

Production of IgG2 antibodies and the activity of cytotoxic T cells are characteristics of Th1 immune response in mice, whereas a predominance of IgM, IgG1, IgA and IgE class antibodies represent Th2 response. In general, vaccines adjuvenated with FCA and delivered subcutaneously generate a Th1 rather than Th2 type immune response [30]. We observed significantly higher titers of *T. evansi* β tubulin specific IgG antibody, specifically of IgG2a isotype, with higher concentrations of TNF-α and lower concentrations of IL-4 in the serum following r*Te-*β-tubulin immunization in mice. Immunized mice demonstrated a progressive drop in serum IL-5 and IL-10 concentrations following challenge. Serum IFN-γ concentration increased in r*Te-*β-tubulin-immunized mice but dropped after 3 days post challenge. Such serum cytokine profiles suggest a strongly Th1 polarized immune response. Mice immunized with r*Te-*β-tubulin also showed falling TNF-α serum concentrations following a brief rise immediately post challenge, suggesting better protection and reduced pathological changes. These results suggest that r*Te-*β-tubulin immunization promotes a Th1-biased recall response following *T. evansi* challenge. Such a Th1 polarized response has been shown to be pivotal for protection against *T. evansi* infections [20–23, 31] as was demonstrated by the longer survival of vaccinates in the present study.

Although the role of adaptive immunity in protection against extracellular pathogens such as trypanosomes are well recognized [32, 33], the independent contributions of the cell-mediated or humoral immune mechanisms are less well understood. Profiles of induced cytokines are, in general, good indicators of the nature of immune responses mounted by the host against a pathogen and may aid in predicting the outcome of infection. Although the levels of various cytokines have served as reliable indicators of the impact of a pathogen on a host, studies [34–36] have shown repeatedly that attempting to predict the outcome of an infection based on a single cytokine response is unlikely to be successful. For example, IFN-γ, an important cytokine of the Th1 subset, activates and stimulates the macrophages to produce IL-12 that not only helps in differentiation of Th1 cells, but also inhibits the expansion of Th2 type T-cell population. IFN-γ also induces class switching to IgG2a [37] and is a hallmark of humoral immunity to trypanosomosis in various natural and experimental hosts [20–23, 31]. Mice immunized with r*Te-*β-tubulin showed higher serum levels of IFNγ following *T. evansi* challenge compared to mice immunized with the adjuvant alone. In the present study, elevated IFN-γ levels may have contributed to the better control of *T. evansi* parasitemia observed in the immunized compared to sham-immunized mice. However, enhanced production of IFN-γ has also been correlated with early mortality in mice infected with *T. congolense* or *T. brucei* [38, 39], primarily owing to the systemic inflammatory response syndrome (SIRS). This was further substantiated by the observation that anti-IFNγ antibodies could prevent early mortality in these infections [40].

The ability of elevated IFNγ levels following virulent challenge with *T. evansi* to both aid control of parasitemia and simultaneously exacerbate damage to the host may be explained by the varied roles that IFN-γ plays in the host. Barkhuizen et al. [41] showed that IFN-γ plays a central role in resistance against trypanosomosis by inducing macrophages to generate trypanocidal molecules such as reactive oxygen intermediates, reactive nitrogen intermediates or TNF-α. Although TNF-α is vital for resistance against trypanosomosis [31], increasing TNF-α levels indicate a failing immune system and an uncontained infection [42–44]. Additionally, it has been challenging to determine if an individual cytokine response is a consequence of an infection or exacerbates one. For example, a surge in IL-10 in serum often follows release of pro-inflammatory cytokines such IFN-γ during an infection; in such cases the surge in IL-10 production is a consequence of inflammation rather than a contributor to it [45]. The cytokines IL-4, IL-5 and IL-10 are thought to balance the pro-inflammatory milieu generated in an infection to keep the host tissue safe from its own inflammatory immune responses. In the protected mice, we observed a spike in serum IFN-γ levels concurrently with lower IL-4 and IL-5 levels immediately after challenge. This IFN-γ surge is followed by a sudden decrease, most likely associated with a control of the infection, as indicated by a corresponding drop of IL-10 and TNF-α concentration in the serum. Although it may be difficult to predict the outcome of an infection from the levels and patterns of individual serum cytokine responses, deducing overall cytokine profiles by measuring multiple, interacting cytokines helps in predicting the course of the infection. This knowledge might also help to define a desirable protective cytokine profile indicative of successful vaccination.

## Conclusions

Mice immunized with r*Te-*β-tubulin in this study showed a predominant Th1 polarization in the elicited immune response as evidenced by both the cytokine profile and the IgG2a isotype of circulating anti-*Te-*β-tubulin antibodies generated in immunized mice. Increased resistance to trypanosomosis in mice may be correlated with higher serum level of the parasite specific IgG2a antibodies; the elevated serum titers of anti-tubulin IgG2a class antibodies post-immunization likely contributed to our observed protection of mice from the lethal challenge. This study reasserts the potential of *prima facie* concealed antigens in eliciting protective humoral immune responses against pathogens; such cryptic antigens may be crucial resources in the fight against complex eukaryotic pathogens and their complicated biologies.

## Abbreviations

°C: Degree celsius
DPC: Day post challenge
ELISA: Enzyme linked immunosorbent assay
FACS: Fluorescence activated cell sorting
FCA: Freund’s complete adjuvant
H: Hour
IFA: Freund’s incomplete adjuvant
IFN-γ: Interferon gamma
IgG2a: Immunoglobulin G 2a isotype
IL-10: Interleukin 10
IL-4: Interleukin 4
IL-5: Interleukin 5
ml: Milliliter
PCR: Polymerase chain reaction
SDS-PAGE: Sodium dodecyl sulphate polyacrylamide gel electrophoresis
*Te-*β-tubulin: *Trypanosoma evansi* beta tubulin
Th1: T-helper cell type 1
Th2: T-helper cell type 2
TNF-α: Tumor necrosis factor alpha
μg: Microgram
μl: Microliter
